# Plumbagin protects rat cortical neurons from mechanical trauma injury-induced apoptosis by inhibiting NOX4/ROS/p38 MAPK pathway

**DOI:** 10.22038/ajp.2025.25704

**Published:** 2025

**Authors:** Qianrui Zhang, Haitan Fu, Wenjuan Gong, Feng Cao, Tao Wu

**Affiliations:** 1 *Department of Pharmacy, General Hospital of the Yangtze River Shipping, Wuhan Brain Hospital, Wuhan, China, 430014*; 2 *Central Laboratory, Wuhan Institute of Clinical Pharmacy* *,* * Wuhan Fourth Hospital, Wuhan, China, 430033*

**Keywords:** Plumbagin, NOX4, TBI, Apoptosis, p38 MAPK

## Abstract

**Objective:**

The aim of this study was to survey whether plumbagin (PLB), a naturally occurring naphthoquinone found in the plants of *Plumbago* genus, can protect rat primary cortical neurons against mechanical injury which classically mimics traumatic brain injury (TBI) *in vitro*.

**Materials and Methods:**

Rat primary cortical neurons were isolated from time-mated pregnant Sprague-Dawley rats and cultured *in vitro*, and then, randomly divided into control group，trauma group，trauma+GKT137831 (50 μM) group，trauma+PLB (5 μM) group, trauma+PLB (10 μM) group and trauma+PLB (20 μM) group．The influence of PLB on rat primary cortical neuron viability and morphology was evaluated after mechanical injury. Flow cytometry was applied to examine neuron apoptotic rate and intracellular production of reactive oxygen species (ROS) after the pretreatment of PLB. The expression of NOX4/p38 MAPK pathway-related proteins was determined by Western blotting.

**Results:**

Our results indicated that PLB pretreatment significantly alleviated trauma induced-neuronal injury by restoring cell viability and reducing lactate dehydrogenase (LDH) leakage compared with the trauma group (p<0.01). The morphology of injured neurons was improved by PLB pretreatment. Also, PLB notably reduced ROS production in cultured rat primary cortical neurons compared with the trauma group (p<0.01). Furthermore, PLB counteracted the mechanical injury-mediated apoptosis (p<0.01) and inhibited the expression of NOX4 protein and p38 MAPK phosphorylation in cortical neurons compared with the trauma group (p<0.01).

**Conclusion:**

The present findings illustrate that PLB can alleviate mechanical trauma injury-induced apoptosis by inhibiting the NOX4/ROS/p38 MAPK pathway in primary cortical neurons.

## Introduction

Traumatic brain injury (TBI) is a serious health problem worldwide due to its high mortality and morbidity. The common reasons of TBI are traffic accident (54%), falls (32-33%) and violence (9-11%) (Maas et al.2017). Based on the different pathophysiological processes, TBI can be divided into primary and secondary cerebral injuries (Li et al. 2015). Primary brain injury is triggered by a direct mechanical force and is usually incurable, whereas, secondary brain injury is induced by molecular events including neuronal apoptosis, oxidative stress, inflammatory reaction, autophagy and so on (Moretti et al. 2015; Schwarzmaier and Plesnila 2014). Secondary injury occurs subsequently after the primary injury, which leads to neurological dysfunctions (Maas et al. 2008). The pathophysiological mechanisms of TBI include excitatory neurotoxicity, mitochondrial dysfunction, modulation of pro-apoptotic factors, overproduction of reactive nitrogen species (RNS) and reactive oxygen species (ROS), and DNA damage (Chiu et al. 2016). Notably increased level of intracellular ROS is one of the key drivers of secondary injury following TBI. During cerebral injury, oxidative stress occurs due to the overproduction of ROS which disrupts the free radical scavenging mechanism. Increased levels of ROS/RNS thus trigger neuron injury through enhancing cell membrane damage, oxidative DNA damage and peroxidation (Prins et al. 2013). Apoptosis of neurons also contributes to the pathological process of secondary cerebral injury during TBI (Omelchenko et al. 2019). Apoptosis of neurons and glial cells is closely associated with the molecular pathological process during TBI in both animals and humans (Uzan et al. 2006). Intracellular calcium, excitatory amino acids and free radicals are responsible for initiating neuronal apoptosis. Cell model studies have demonstrated that neurons experience apoptosis via a series of intracellular signaling (Eldadah and Faden 2000). Mitogen-activated protein kinases (MAPKs) have been shown to play vital roles in cellular signal modulation during TBI (Kim and Choi 2010). Various stress factors including chemical signals and physical changes can stimulate p38, JNK and ERK, thus initiating the Raf/MEK/ERK signal cascade via G protein-coupled receptors. Among them, the p38 MAPK pathway has been intensively investigated and confirmed to play a vital role in the molecular pathological processes of TBI, which makes it an attractive target of intervention (Li et al. 2022).

Plumbagin (5-hydroxy-2-methyl-1,4-naphthoquinone, PLB), a quinoid compound isolated from the medicinal herb of *Plumbago zeylanica* L. (Plumbaginaceae) which is traditionally used in traditional Chinese medicine especially for cancer (Chen et al. 2011), has been described to modulate inflammatory activation and redox status against experimental neurotoxicity (Kuan-Hong and Bai-Zhou 2018; Wang et al. 2018). The content of PLB is much higher in root than in other parts (leaves and stems) of *P. zeylanica* L. Thus, the root of *P. zeylanica* L is likely the most effective medicinal part and a major source of PLB (Jaradat et al. 2021). According to our previous experiment, PLB could effectively relieve oxygen-glucose deprivation/reoxygenation-induced neuronal injury by targeting NLRP3 inflammasome activation *in vitro* (Zhang et al. 2020). Also, in animal experiments, PLB has been demonstrated to inhibit neuronal apoptosis in rat cerebral ischemia, which might be due to its suppression of TNF-α/NF-κB pathway (Chen et al. 2018). The balance between pro-apoptotic proteins (Bax and Bad) and the anti-apoptotic proteins (Bcl-2 and Bcl-xL), predominantly regulates the mitochondrial membrane integrity and the release of cytochrome C and apoptogenic factors that influence cell death (Zhao et al. 2003). Interestingly, prior treatment with PLB obviously increased the expression of Bcl-2 and Bcl-xL while down-regulating Bax and Bad and cleaved caspase-3, which might contribute to its anti-apoptotic effect on ischemic neurons. These aforementioned studies suggest that PLB may be used therapeutically to improve central nervous system (CNS) diseases. 

Therefore, we examined the hypotheses that PLB is neuroprotective in an *in vitro* model of TBI, and that the NOX4/ROS/p38 MAPK pathway is involved in mediating PLB-induced neuroprotectionin rat primary cortical neurons. This is the first study to evaluate the potential of PLB for improvement of TBI in cell model and to explore its mechanism of action simultaneously.

## Materials and Methods

### Reagents

PLB (Cat. No. S4777) and GKT-137831 (Cat. No. S7171) were obtained from Selleck (Houston, USA). The MAP-2 (Cat. No. PAB33401), NeuN (Cat. No. MAB46104), p38 MAPK (Cat. No. PAB38871) and NOX4 (Cat. No. PAB30655) antibodies were obtained from Bioswamp (Wuhan, China). The primary antibodies against phospho-p38 MAPK (Cat. No. 4511T) and cleaved caspase 3 (Cat. No. 9661T) were obtained from CST (Danvers, MA, USA). The primary antibody against GAPDH was purchased from Abcam (Cambridge, UK).

### Preparation of primary rat cortical neurons

All procedures applied on rats were authorized by the Ethical Committee on Animal Experimentation of Wuhan Fourth Hospital (ethical approval number: No. 2018-14, Wuhan, China) and followed The National Institutes of Health Guidelines on the Use of Laboratory Animals. In the present study, time-mated pregnant Sprague-Dawley rats (SPF class, n=3) were adopted. Rat primary cortical neurons were prepared as previously described (Carey et al. 2002). Briefly, pregnant rats were euthanized with sodium pentobarbital (150 mg/kg, intraperitoneal injection (i.p.)) at 16–17 days' gestation. Animal death was confirmed by cardiac arrest. After that, the fetuses were decapitated and the cortices were isolated rapidly. The cerebral cortex was treated with 0.25% trypsin and 0.01% deoxyribonuclease I for 15 min at 37˚C. Then, the neurons were dissociated mechanically and cultured in neurobasal medium (Gibco, Thermo Fisher Scientific, Inc.). The cortical neurons were plated at a density of 7x10^5^ cells in 12-well plates and maintained at 37˚C in humidified 5% CO_2_ and 95% air. On the next day, culture medium was changed to serum-free neurobasal medium (45 ml) containing B27 (5 ml) and 1% penicillin-streptomycin. Half of the medium was changed every 3 days. Cultured neurons were used at 12-14 days and confirmed to be >95% neurofilament-positive by immunostaining.

### Immunofluorescence

Cells were cultured on coverslips coated with lysine. After being fixed in 4% paraformaldehyde at room temperature for 30 min, neurons were washed twice with phosphate buffer saline (PBS), then treated with primary antibodies (anti-NeuN (1:2,00, Bioswamp, MAB46104) and anti-MAP-2 (1:2,00, Bioswamp, PAB33401)) overnight at 4˚C, respectively. After that, the slices were processed by corresponding secondary antibody (1:2,00, Bioswamp, SAB43732). The fluorescence was photographed through a fluorescence microscope (DMIL LED; Leica Microsystems GmbH).

### In vitro TBI model

A mechanical trauma injury cell model mimicking TBI was constructed as previously reported (Zhou et al. 2014). In brief, each 35-mm dish confluent culture was scratched by using a sterile 21-gauge needle following a 9x9 square grid (4 mm spacing between the lines). The scratches induced immediate cell death under the blade followed by secondary injury to surrounding neurons. Measurements were performed a day after injury. Uninjured cultures were set as the control group. To investigate the protective activities of PLB, cultured neurons were pretreated with PLB (5, 10 or 20 μM) or GKT-137831 (50 μM) respectively before scratch injury for 6 hr.

### Cell viability

 Viability of neurons was tested with commercially available MTT kit (Cat. No. M1025) in accordance with the manufacturer’s guidance (Solarbio, Beijing, China). The neurons were cultured in a 96-well plate following treatments at 37˚C. After which, MTT solution (10 µl, 5 mg/ml) was added at 24 hr, followed by incubation for 4 hr. Results were obtained by detecting the absorbance at 490 nm via a microplate reader (Hangzhou Allsheng Instruments Co., Ltd.).

### Lactate dehydrogenase (LDH) assays

LDH release into the culture medium was measured 24 hr after injury using a commercially available LDH kit (Cat. No. A020-2-1) in accordance with the manufacturer’s instructions (Jiancheng, Nanjing, China). The absorbance was determined at 490 nm via a microplate reader (Hangzhou Allsheng Instruments Co., Ltd.).

### Apoptosis assessment

After different treatments, cells were gathered and washed with PBS twice. The apoptotic rate was determined with an Annexin V-FITC/PI apoptosis kit (Cat. No. 556547; BD Biosciences). Neuron density was adjusted to 1x10^6^ cells/ml by binding buffer. After which, neurons were marked with 10 µl Annexin V-FITC and 10 µl PI, followed by incubation at 4˚C for 30 min. Then, apoptosis detection was conducted with a flow cytometer (ACEA NovoCyte; ACEA Bioscience, Inc.). Results were analyzed by NovoExpress 1.5 (ACEA Bioscience, Inc.).

### ROS assessment

ROS levels were estimated by detecting 2',7'‑dichlorodihydrofluorescein (DCFH)-derived fluorescence by flow cytometry. Neurons were incubated with 10 µM DCFH-diacetate (Sigma-Aldrich) in serum‑free Dulbecco’s Modified Eagle’s Medium (DMEM) for 20 min at 37˚C under 5% CO_2_ (1.0x10^6^/ml). After which, neurons were gathered and suspended in 500 µl PBS. ROS levels were evaluated by measuring DCF fluorescence with a flow cytometer (ACEA NovoCyte; ACEA Bioscience, Inc.). Results were analyzed by NovoExpress 1.5 (ACEA Bioscience, Inc.).

### Western blotting

Neurons from different groups were washed and lysed in radio immunoprecipitation assay (RIPA) lysis buffer (Cat. No. R0010; Solarbio, Beijing, China) for 30 min on ice, and centrifuged for 10 min (4˚C, 12000 g). The protein concentration in the supernatant was assessed by a commercial BCA Kit (Cat. No.PC0020; Solarbio, Beijing, China). The sample (20 μg) was resolved by SDS-PAGE and blotted electrophoretically onto a PVDF membrane, then blocked with 5% non-fat milk followed by incubation with the following primary antibodies: Anti-NOX4 (1:1,000), anti-cleaved caspase 3 (1:1,000), anti-p38 MAPK (1:1,000), anti-phospho-p38 MAPK (1:1,000) and anti-GAPDH (1:1,000) overnight at 4˚C. After incubation with corresponding secondary antibodies, proteins were visualized and quantified by Tanon GIS software (Ver.4.00, Tanon Science and Technology Co., Ltd.).

### Statistical analysis

All data are expressed as mean ± SD and were analyzed by SPSS software (version 18.0; SPSS, Inc.). One-way analysis of variance followed by Tukey's post hoc test was conducted for data comparisons. Differences with p<0.05 was considered significant statistically and differences with p<0.01 was considered to be extremely statistically significant.

## Results

**PLB prevents** **mechanical trauma induced neuronal death**

**Figure 1:**
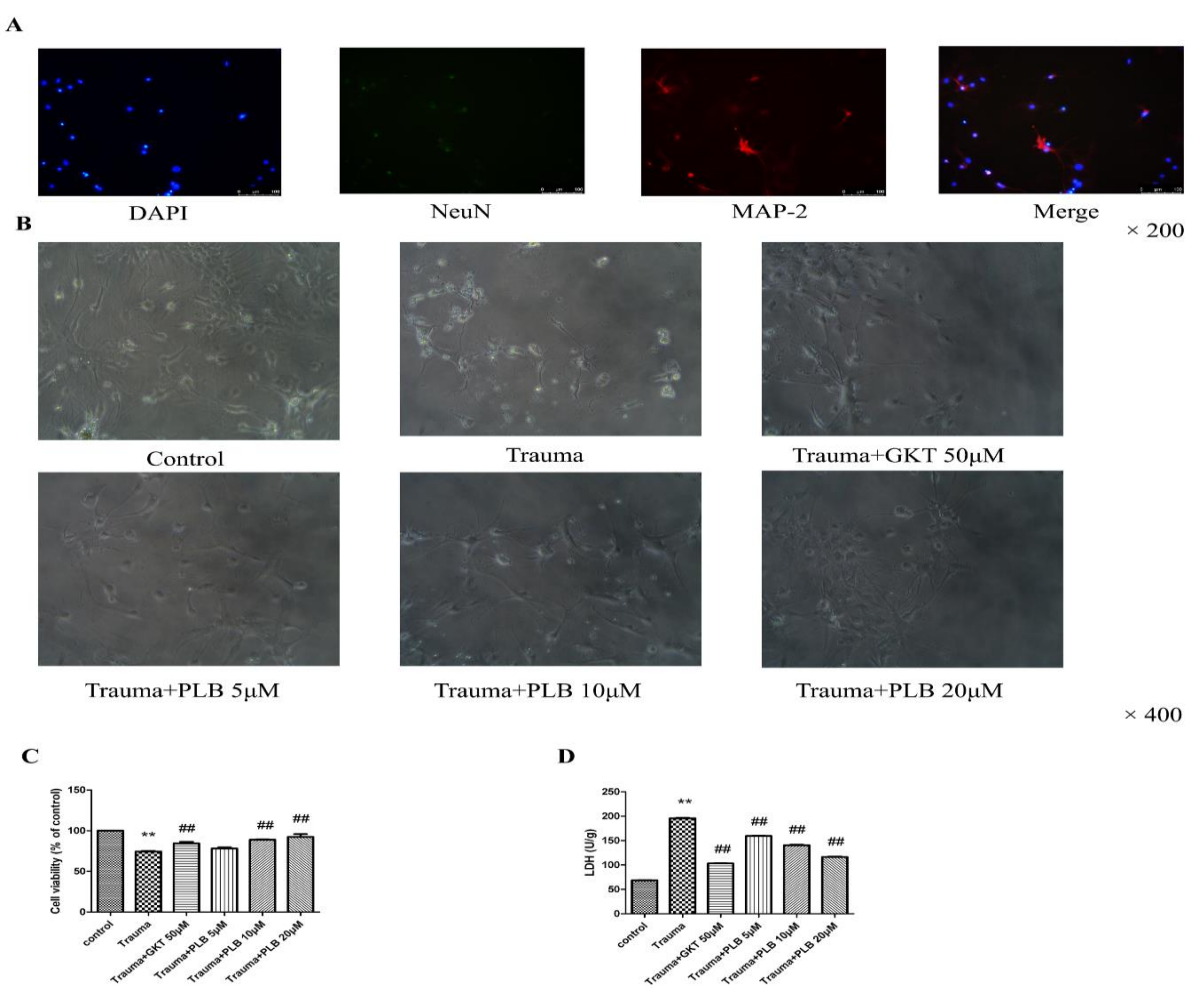
Effects of PLB on cell death induced by mechanical trauma. (A) Representative confocal images of cultured primary neurons showing immunoreactivity to NeuN (green) and MAP-2 (red). Nuclei stained with DAPI (blue) (magnification, x200). (B) Morphology of cultured cortical neurons (magnification, x400). (C) Cell viability of neurons was measured by MTT assay (n=6). (D) LDH activity (n=6). The results are presented as the means ± S.D. *p<0.05 versus control, **p<0.01 versus control; #p<0.05 versus trauma, and ##p<0.01 versus trauma as assessed by one-way ANOVA with Tukey’s post hoc analysis. PLB, plumbagin.

To evaluate the protective activity of PLB, cultured neurons were pretreated with PLB for 6 hr before mechanical trauma. The morphology of cultured cortical neurons was evaluated under inverted microscope, cell viability was determined by MTT method and neuronal death was estimated via the detection of LDH activity in the medium. Immunocytochemistry of cultured primary neurons is shown in Figure 1A (magnification, x200). As shown in Figure 1B, the morphology of mechanical injury-induced neurons was improved by PLB pretreatment or GKT pretreatment. As shown in Figure 1C, mechanical injury notably repressed neuron viability compared with the control group, indicating obvious cytotoxicity. PLB (10 and 20 μM) pretreatment notably restored trauma-decreased neuron viability compared with the trauma group. As shown in Figure 1D, an obvious elevation in LDH activity was demonstrated at 24 hr after scratch injury (p<0.01). And PLB (5, 10 and 20 μM) pretreatment notably reduced mechanical injury-induced LDH activity (p<0.01). Furthermore, GKT (50 μM) pretreatment increased cell viability and reduced LDH activity compared with the trauma group (p<0.01).

**PLB reduces** **mechanical trauma-induced ROS production**

**Figure 2:**
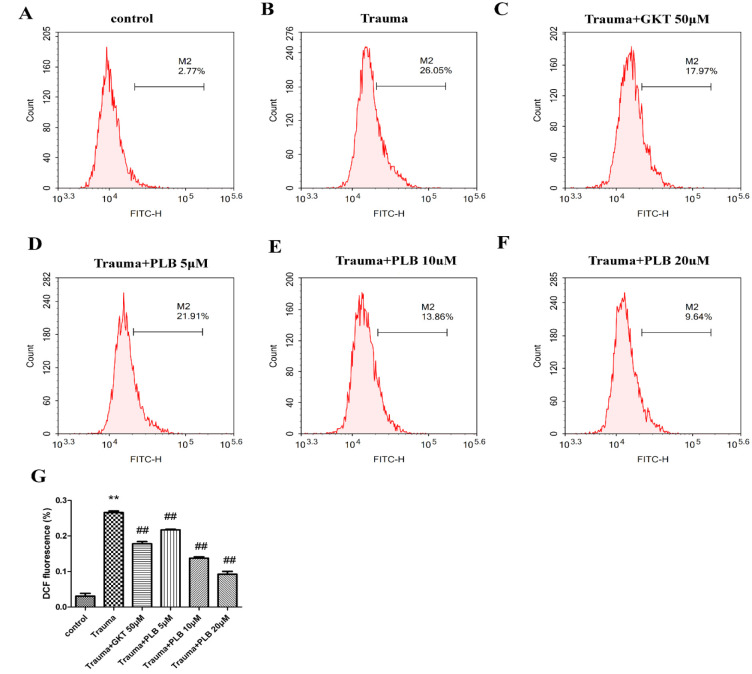
Effects of PLB on the elevation of ROS induced by mechanical trauma. (A) Control group, (B) trauma group, (C) trauma + GKT (50 μM), (D) trauma + PLB (5 μM), (E) trauma + PLB (10 μM) and (F) trauma + PLB (20 μM). (G) Bar graph presents DCF fluorescence intensity of the different groups. The data were obtained from at least three independent experiments. The results are presented as the means ± S.D. *p<0.05 versus control, **p<0.01 versus control; #p<0.05 versus trauma, and ##p<0.01 versus trauma as assessed by one-way ANOVA with Tukey’s post hoc analysis. PLB, plumbagin; ROS, reactive oxygen species.

The ROS level in primary rat cortical neurons was evaluated by DCFH-DA staining. The DCF fluorescence intensity reflects the intracellular ROS level. As shown in Figure 2A-G, the DCF fluorescence intensity in the trauma group was markedly increased compared with the control group (p<0.01), indicating elevated ROS levels. Furthermore, DCF fluorescence intensity in the trauma+PLB groups was remarkably decreased compared with the trauma group (p<0.01). Moreover, GKT (50 μM) pretreatment notably reduced DCF fluorescence intensity compared with the trauma group (p<0.01).

**PLB alleviates neuronal apoptosis induced by mechanical trauma**

**Figure 3:**
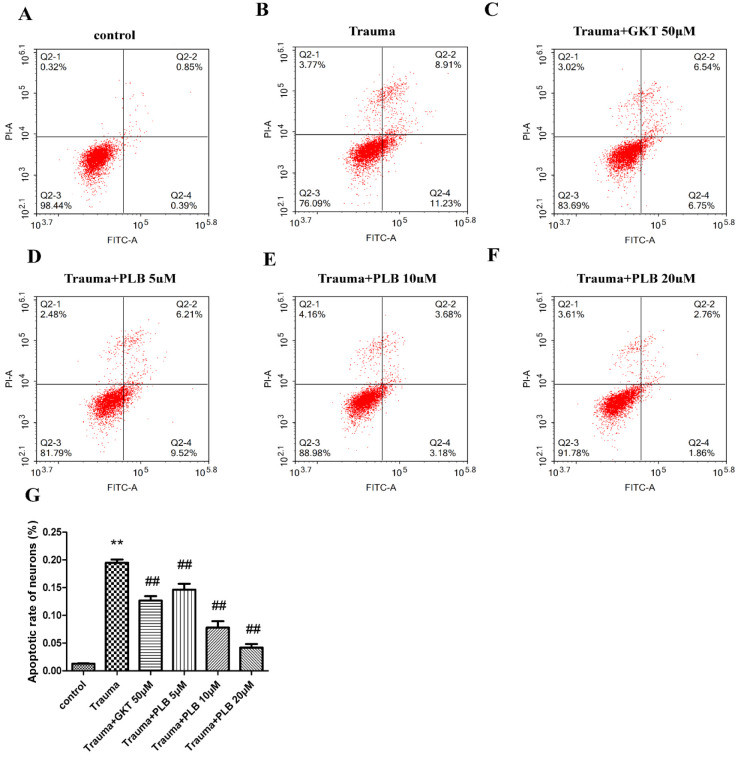
Effects of PLB on cell apoptosis induced by mechanical trauma. (A) Control group, (B) trauma group, (C) trauma + GKT (50 μM), (D) trauma + PLB (5 μM), (E) trauma + PLB (10 μM) and (F) trauma + PLB (20 μM). (G) Bar graph presents apoptotic rates of the different groups. The data were obtained from at least three independent experiments. The results are presented as the means ± S.D. *p<0.05 versus control, **p<0.01 versus control; #p<0.05 versus trauma, and ##p<0.01 versus trauma as assessed by one-way ANOVA with Tukey’s post hoc analysis. PLB, plumbagin.

To confirm apoptosis, the neurons were stained with Annexin V and PI. After which, flow cytometry was conducted to determine apoptotic neurons. As shown in Figure 3A-G, the apoptotic rate was notably elevated in the trauma group compared with the control group (p<0.01). Besides, the apoptotic rate in the trauma+PLB groups was notably decreased compared with the trauma group (p<0.01). Also, pretreatment with GKT (50 μM) markedly decreased the apoptotic rate compared with the trauma group (p<0.01). 

**PLB suppresses the NOX4/p38 MAPK pathway induced by mechanical trauma**

**Figure 4:**
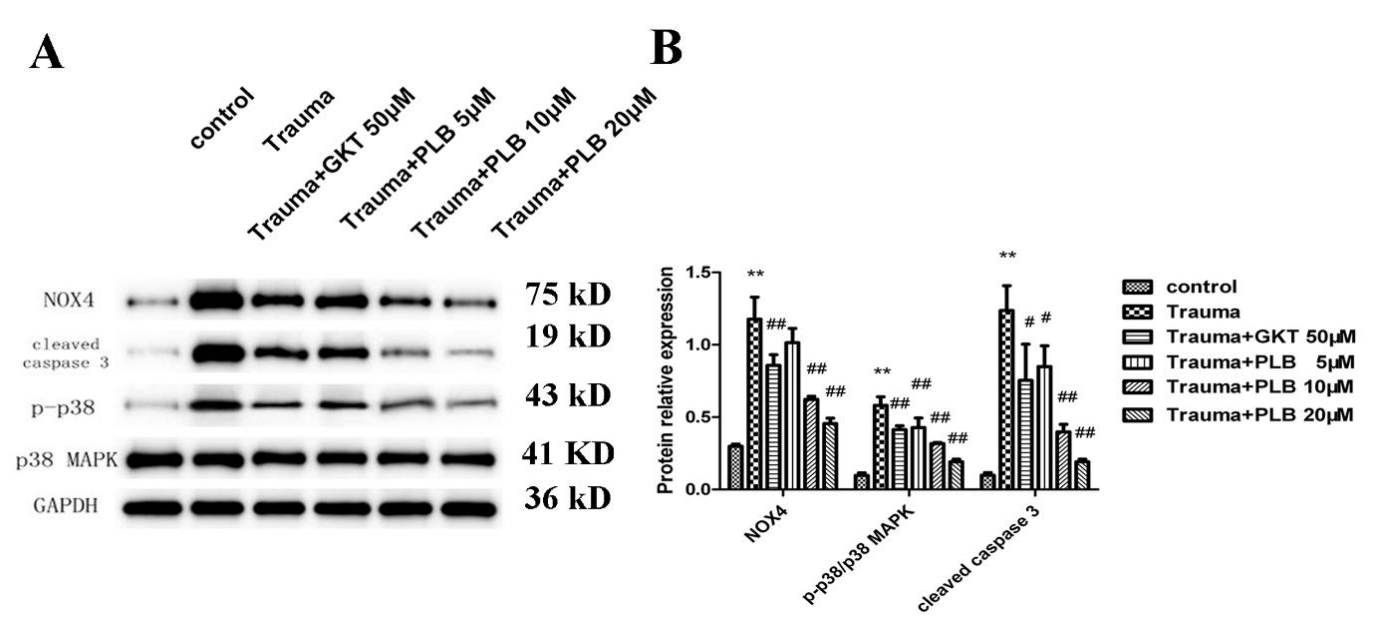
Effects of PLB on the NOX4 and apoptotic protein expression induced by mechanical trauma. (A) Protein expression levels of NOX4, cleaved caspase 3 and phospho-p38/p38 MAPK were examined by western blot analysis. GAPDH was used as a loading control. (B) Quantitative analysis of NOX4, cleaved caspase 3 and phospho-p38/p38 MAPK protein expression. The data were obtained from at least three independent experiments. Bars represent means ± S.D. *p<0.05 versus control, **p<0.01 versus control; #p<0.05 versus trauma, and ##p<0.01 versus trauma as assessed by one-way ANOVA with Tukey’s post hoc analysis. PLB, plumbagin.

The expression of phospho-p38/p38 MAPK, cleaved caspase 3 and NOX4 proteins in the trauma group was markedly up-regulated compared with the control group (p<0.01). Pretreatment with PLB (10 and 20 μM) or GKT (50 μM) significantly suppressed NOX4 expression compared with the trauma group (p<0.01). In addition, PLB (5, 10 and 20 μM) or GKT (50 μM) pretreatment significantly suppressed cleaved caspase 3 expression compared with the trauma group (p<0.05 and p<0.01). Furthermore, PLB (5, 10 and 20 μM) or GKT (50 μM) pretreatment significantly inhibited phospho-p38/p38 MAPK expression compared with the trauma group (p<0.01; Figure 4).

## Discussion

As a secondary metabolite obtained from different plant families such as Plumbaginaceae, Droseraceae, and Ebenceae, PLB has been reported to exert beneficial effects on different human diseases including cancer, diabetes, malaria, and bacterial infection as well as cardiovascular disease (Thakor and Janathia 2022). Recently, its therapeutic potential on CNS diseases has become a new research hotspot (Santos et al. 2023). There are no officially approved therapeutic modalities available despite extensive studies have been conducted on TBI. It is necessary to develop novel therapeutic 

agents (Kaur and Sharma 2018; Capizzi et al. 2020). PLB has been previously reported to alleviate experimental memory and behavior deficits induced by intrahippocampal injection of quinolinic acid *in vivo*, suggesting its neuroprotective effect (Kumar et al. 2022). Our data presented in this research demonstrated that PLB ameliorated mechanical trauma-induced neuronal injury by inhibiting the p38 MAPK signaling pathway. One of our most notable results was the neuroprotective activity of PLB on TBI by inhibiting neuronal apoptosis, as demonstrated by a reduction of the apoptotic rate in neurons. Furthermore, pretreatment of GKT137831, a NOX4 selective inhibitor, protected neurons against mechanical trauma-induced apoptosis and repressed p38 MAPK activation, which might suggest the vital role of NOX4 in TBI pathogenesis. 

Oxidative stress involves the imbalance between pro-oxidants and antioxidants and the overproduction of free radicals (such as ROS and RNS) in response to TBI (Rodríguez-Rodríguez et al. 2014). ROS and RNS are generated by kinds of pathways activated following TBI. Those free radicals participate in the pathological processes of TBI by disrupting cerebrovascular tissues through membrane lipid peroxidation. Lipid peroxidation is a common oxidative damage mechanism that occurs in the brain during TBI. The high content of lipid and abundance of iron in the brain make neuronal membranes at a high risk of lipid peroxidative injury (Lewén et al. 2000). This process triggers vascular system peroxidation, protein oxidation, inhibition of the mitochondrial electron transport chain and DNA damage. Then, neuroinflammation, neuron death and apoptosis are activated seriously (Khatri et al. 2018). NADPH-oxidase (NOX), especially NOX4, is responsible for the production of ROS during TBI, and is the only enzyme that solely functions to generate ROS (Altenhöfer et al. 2012). It should be noted that, in the acute phase of TBI, serum NOX4 levels could reflect severity directly, suggesting the key role of NOX4 in the pathogenesis of TBI (Jiang et al. 2022). The role of NOX4 in CNS diseases has already been definitely illustrated in recent years. For instance, NOX4 in the hippocampus has been proved to generate massive ROS and cause a redox imbalance, thus contributing to the neurotoxicity of Parkinson's disease (PD) in mouse model (Boonpraman et al. 2023). In intracerebral hemorrhage (ICH) model, the upregulation of NOX4 was found to elevate the level of oxidative stress, increase hematoma volume and exacerbate brain edema (Liao et al. 2024). Furthermore, NOX4 knockout can ameliorate tissue damage, oxidative stress injury, neurodegeneration and neuronal death after focal TBI in mice (Ma et al. 2018). Also, our study demonstrated that PLB or GKT137831 could inhibit the mechanical trauma-induced NOX4 expression and neuronal injury *in vitro*. Therefore, PLB might protect post-injury oxidative damage following TBI via the repression of NOX4. 

Apoptosis of glia and neurons is a key contributor to the TBI pathology in animals and humans (Keane et al. 2001). Elevation in the levels of excitatory amino acids, intracellular calcium and free radicals can drive neurons to initiate apoptosis (Payette et al. 2008). Studies have revealed that the activation of cysteine proteinases (caspases 1-9) is involved in the molecular basis of cell apoptosis (Springer et al. 2001; Xu et al. 2024; Binjawhar et al. 2024). When TBI occurs, an apoptotic enzyme cascade is activated, including the initiator caspases (caspases 8 and 9) which are stimulated at the initial stage of the apoptotic signaling cascade, and the effector caspases (caspases 3, 6 and 7) which are later activated. The activated effector caspases can cleave numerous nuclear and cytoplasmatic proteins and stimulate DNA-cleaving endonucleases with caspase 3 being an end point of the apoptotic signaling in the brain (Dressler et al. 2007). Our results in this study indicated that PLB could inhibit caspase 3 activation in mechanical trauma-induced neurons, thus contributing to the alleviation of apoptosis.

Considerable evidence demonstrates that the p38 MAPK pathway is obviously activated following TBI (Zhao et al. 2022). In brain tissue, the p38α and p38β are abundantly expressed and are regularly activated under inflammatory conditions during TBI, leading to activation of response genes, changes in physiological characteristics and neurotoxicity (Wei and Hsieh 2020). Thus, the p38 MAPK subtypes play a vital role in the pathological mechanism of TBI, making it an attractive target of intervention (Lan et al. 2019). On the other hand, NOX4 overexpression has been demonstrated to notably activate ROS production and p38 MAPK expression *in vitro* (Goettsch et al. 2009). The subsequent influences of p38 MAPK activation depend on the accumulation of ROS generated by NOX4 activity (Tormos et al. 2013; Yi et al. 2019) have confirmed that NOX4 repression could decrease uranium-induced p38 MAPK activation in hepatocytes, suggesting that NOX4-mediated ROS generation could be closely associated with p38 MAPK activation. In this study, pretreatment with GKT137831 not only repressed the up-regulation of NOX4 expression, but also reduced p38 MAPK phosphorylation in mechanical trauma-induced neurons, revealing that NOX4-mediated ROS production could be closely related to the mechanical trauma-stimulated p38 MAPK phosphorylation in neurons. Furthermore, PLB reduced p38 MAPK phosphorylation and NOX4 expression, suggesting that PLB might protect mechanical trauma-induced neuron injury, at least partially, through the NOX4/p38 MAPK pathway.

To summarize, we found that the anti-apoptotic and neuroprotective activities of PLB in an *in vitro* TBI model might be due to the repression of the NOX4/ROS/p38 MAPK pathway. This study has a few limitations, however. The role of other cellular signals in the neuroprotective activity of PLB cannot be excluded due to the complex pathological processes and the interactions of the p38 MAPK pathway with other molecular signals. Also, the result of this *in vitro* experiment might not fully reflect the action of PLB *in vivo*. Thus, the related animal research is needed to be conducted in next. 

## Data Availability

All data in this study are available upon request by contact with the corresponding author.
